# Maladie de Von Recklinghausen compliquée de Neurofibromes plexiformes cervico-faciaux

**DOI:** 10.11604/pamj.2015.20.408.6122

**Published:** 2015-04-27

**Authors:** Madiha Mahfoudhi, Khamassi Khaled

**Affiliations:** 1 Service de Médecine Interne A, Hôpital Charles Nicolle, Tunis, Tunisie; 2 Service d'ORL, Hôpital Charles Nicolle, Tunis, Tunisie

**Keywords:** Maladie de Von Recklinghausen, neurofibrome, plexiformes cervico-faciaux, Von Recklinghausen disease, neurofibroma, cervico-facial plexiformsx

## Image en medicine

La neurofibromatose de type 1 (NF1), appelée également maladie de Von Recklinghausen, est une affection autosomique dominante caractérisée par son polymorphisme clinique. Les neurofibromes dans le cadre de cette maladie peuvent être cutanés, sous-cutanés ou plexiformes. Les neurofibromes plexiformes siègent surtout au niveau des paupières, du tronc et des membres. La localisation dans la région cervico-faciale est rare. L'examen clinique et l'imagerie, sont souvent suffisants pour poser le diagnostic. L’évolution est imprévisible justifiant une surveillance clinique et radiologique prolongée. Patient âgé de 21 ans, suivi pour une neurofibromatose de Von Recklinghausen, a consulté pour une tuméfaction de l'hémiface gauche de 5 cm de grand axe. L'examen a révélé une hypoplasie de la mandibule, des os frontal et temporal gauches, une oreille gauche bas implantée. A la palpation, il avait deux tuméfactions pigmentées de consistance molle: la première étendue depuis la loge parotidienne vers la loge submandibulaire, et la deuxième siégeant au niveau de la conque et comblant le méat auditif externe. A la tomodensitométrie cervico-faciale, il avait un volumineux processus expansif de 11x10x5 cm centré sur la loge parotidienne gauche, avec une parotide complètement envahie par ce processus. Cette masse infiltre le méat auditif externe en dehors et les muscles ptérygoïdiens et la graisse parapharyngée en dedans. Cet aspect était évocateur d'un neurofibrome plexiforme. Il a bénéficié d'une plastie du pavillon gauche. L’évolution était marquée par la stabilisation de la taille de la tumeur faciale.

**Figure 1:**
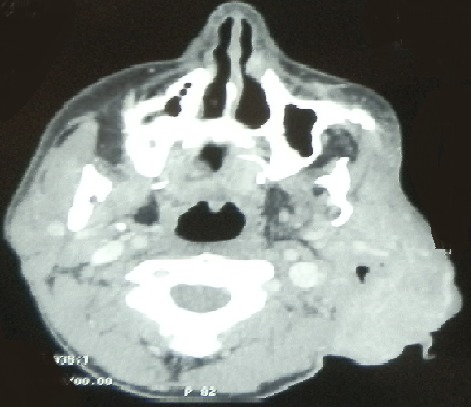
TDM cervico-faciale (coupe axiale): processus expansif centré sur la loge parotidienne gauche

